# The first complete mitochondrial genome of Melongenidae from *Hemifusus tuba* (Neogastropoda: Buccinoidea)

**DOI:** 10.1080/23802359.2019.1674746

**Published:** 2019-10-09

**Authors:** Shengping Zhong, Lianghua Huang, Guoqiang Huang, Yonghong Liu, Weiling Xu

**Affiliations:** a Institute of marine drugs, Guangxi University of Chinese Medicine, Nanning, Guangxi, China;; b Key Laboratory of Marine Biotechnology, Guangxi Institute of Oceanology, Beihai, Guangxi, China;; c Institute of Economic Management, Beihai Vocational College, Beihai, Guangxi, China

**Keywords:** Mitochondrial genome, *Hemifusus tuba*, Neogastropoda

## Abstract

*Hemifusus tuba* is an ecologically and economically important species of Buccinoidea, which comprises an ecologically diverse group of carnivorous marine gastropods. However, the taxonomic classification and phylogenetic studies have so far been limited. In this study, we report the first complete mitochondrial genome of Melongenidae from *H. tuba*. The mitogenome has 15,483 bp (68.2% A + T content) and made up of total of 37 genes (13 protein-coding, 22 transfer RNAs, and 2 ribosomal RNAs), and a control region. This study was the first to provide complete mitogenome of Melongenidae and will provide useful genetic information for future phylogenetic and taxonomic classification of Buccinoidea.

The Buccinoidea are one of the most geographically widespread and ecologically diverse clade within the Neogastropoda, which comprise about 1000 described species and inhabited all over the world oceans from tropical oceans to the poles and from the intertidal to the deep seas (Kantor 2003). The family Melongenidae is a small family of Buccinoidea, which generally divided into two subfamilies (Hayashi 2005). Many species of Melongenidae including *Hemifusus tuba* are economically important as luxury seafood and valuable ingredients of traditional medicines (Li et al. 2019). However, the taxonomy and phylogeny of the Buccinoidea have been debated due to lack of reliable morphological taxonomic characters (Kantor 2003). The complete mitochondrial genome is an excellent molecular marker for studying phylogenetic relationships and taxonomy identification, but adequate mitogenome information about the Melongenidae is still missing. Here, we report the first complete mitochondrial genome sequence of Melongenidae, which will provide a better insight into phylogenetic assessment and taxonomic classification.

A tissue samples of *H. tuba* from 5 individuals were collected from Guangxi province, China (Beihai, 21.023316 N, 109.123369 E), and the whole body specimen (#GR0215) were deposited at Marine Biological Herbarium, Guangxi Institute of Oceanology, Beihai, China. The total genomic DNA was extracted from the muscle of the specimens using an SQ Tissue DNA Kit (OMEGA, Guangzhou, China) following the manufacturer’s protocol. DNA libraries (350 bp insert) were constructed with the TruSeq Nano^TM^ kit (Illumina, San Diego, CA) and were sequenced (2 × 150 bp paired-end) using HiSeq platform at BGI Company (Shenzhen, Guangdong, China). Mitogenome assembly was performed by MITObim (Hahn et al. 2013). The complete mitogenome of *Buccinum pemphigus* (GenBank accession number: NC_029373) was chosen as the initial reference sequence for MITObim assembly. Gene annotation was performed by MITOS (Bernt et al. 2013).

The complete mitogenome of *H. tuba* was 15,483 bp in length (GenBank accession number: MN462591), and containing the typical set of 13 protein-coding, 22 tRNA and 2 rRNA genes, and a putative control region. The overall base composition of the mitogenome was estimated to be A 30.2%, T 37.9%, C 15.6%, and G 16.2%, with a high A + T content of 68.2%, which is similar, but slightly higher than *Indothais lacera* (68.1%) (Zhong et al. 2017). The mitogenomic phylogenetic analyses showed that family Melongenidae was clustered between family Buccinidae and family Nassariidae within superfamily Buccinoidea clade with high bootstrap value (Figure 1), which is consistent with the phylogenetic analyses of Buccinoidea using nuclear gene (*18S rRNA* and segment histone H3) and mitochondrial gene (*COI*, *12S rRNA*, and 1*6S rRNA*) (Zou et al. 2011). The complete mitochondrial genome sequence of *H. tuba* was the first sequenced mitogenome in Melongenidae, which will contribute to further phylogenetic and comparative mitogenome studies of Melongenidae, and related families.

**Figure 1.**
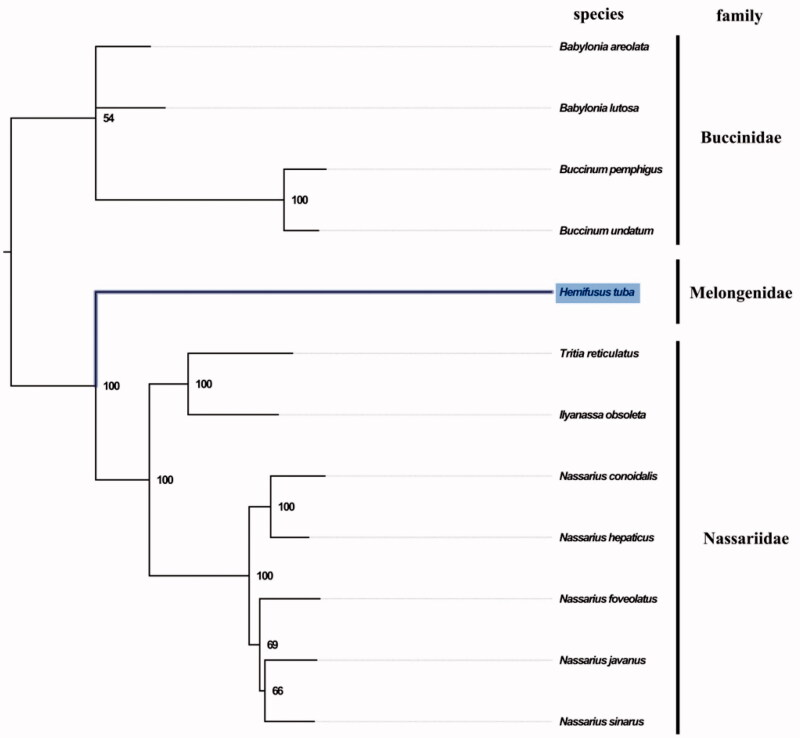
Phylogenetic tree of 12 species in order Neogastropoda. The complete mitogenomes are downloaded from GenBank and the phylogenic tree is constructed by maximum-likelihood method with 100 bootstrap replicates. The bootstrap values were labelled at each branch nodes. The gene’s accession number for tree construction is listed as follows: *Babylonia areolata* (NC_023080), *Babylonia lutosa* (NC_028628), *Tritia reticulatus* (NC_013248), *Rapana venosa* (NC_007781), *Nassarius conoidalis* (NC_041310), *Nassarius hepaticus* (NC_038169), *Nassarius foveolatus* (NC_041546), *Nassarius javanus* (NC_041547), *Nassarius sinarus* (NC_041545), *Buccinum pemphigus* (NC_029373), and *Buccinum undatum* (NC_040940).
